# Reply to Small, K.W. Comment on “Spedicati et al. Hidden in the Genome: The First Italian Family with North Carolina Macular Dystrophy Carrying a Novel *PRDM13* and *CCNC* Duplication. *Biomedicines* 2025, *13*, 1904”

**DOI:** 10.3390/biomedicines14061227

**Published:** 2026-05-29

**Authors:** Beatrice Spedicati, Domizia Pasquetti, Aurora Santin, Stefania Zampieri, Anna Morgan, Stefania Lenarduzzi, Giuseppe Giovanni Nardone, Elisa Paccagnella, Stefania Cappellani, Laura Diplotti, Stefano Pensiero, Fulvio Parentin, Paolo Gasparini, Maurizio Battaglia Parodi, Giorgia Girotto

**Affiliations:** 1 Department of Medicine, Surgery and Health Sciences, University of Trieste, 34149 Trieste, Italypaolo.gasparini@burlo.trieste.it (P.G.);; 2 Institute for Maternal and Child Health—IRCCS “Burlo Garofolo”, 34137 Trieste, Italy; domizia.pasquetti@burlo.trieste.it (D.P.); stefania.zampieri@burlo.trieste.it (S.Z.); anna.morgan@burlo.trieste.it (A.M.); stefania.lenarduzzi@burlo.trieste.it (S.L.); giuseppegiovanni.nardone@burlo.trieste.it (G.G.N.); elisa.paccagnella@burlo.trieste.it (E.P.); stefania.cappellani@burlo.trieste.it (S.C.); laura.diplotti@burlo.trieste.it (L.D.); stefano.pensiero@burlo.trieste.it (S.P.); fulvio.parentin@burlo.trieste.it (F.P.); 3 Ophthalmology Department, Vita-Salute San Raffaele University, 20132 Milan, Italy; battagliaparodi.maurizio@hsr.it

Thank you for sharing the comment [1] on our article “Hidden in the Genome: The First Italian Family with North Carolina Macular Dystrophy Carrying a Novel *PRDM13* and *CCNC* Duplication” [2].

As usual, we appreciate comments from the Readers and are open to any change and or revision; indeed, the comment provided by Dr. Small added a useful addition to the evolving NCMD literature.

Nevertheless, in this case, it is quite difficult to understand the goal of the reader and what they are asking for. As a matter of fact, Dr. Small suggested modifying the title of our paper and questioned the originality of our work based on the assumption that one of the two French families described in the manuscript by Manes et al. [3] could be of Northern Italian descent. Unfortunately, as noted in the comment itself, this information was not included in the original publication nor included in any following publication; therefore, it was not available (nor could it be) in the literature during the preparation of our manuscript. Scientific literature must be grounded in verifiable, published evidence rather than speculative information. Consequently, we welcome this opportunity to affirm the originality and scientific rigour of our work.

In our study, the designation “Italian” of our family refers to (a) clinical ascertainment in Italy, (b) documented Italian ancestry (pedigree up to the fourth generation), and (c) molecular ancestry using PCA (principal component analysis). Therefore, the term “Italian family” in our manuscript reflects both the geographic ascertainment and the demonstrated genetic origin of the family studied.

In conclusion, the lack of any other Italian family described so far in the literature and the strong evidence of the Italian descent in our case prompted us to refer to the geographic origin in the title of the manuscript, which we believe still remains appropriate. 

We thank the Author of the comment and the Editors for fostering constructive academic dialogue.
